# Early therapeutic drug monitoring of methotrexate and its association with acute kidney injury: A retrospective cohort study

**DOI:** 10.1002/cam4.70176

**Published:** 2024-09-10

**Authors:** Nicolás Tentoni, Miriam Hwang, Gabriela Villanueva, Ryan Combs, Jennifer Lowe, Laura B. Ramsey, Zachary L. Taylor, Thais Murciano Carrillo, María Dolores Aumente, Teresa López‐Viñau López, Carmelo Rizzari, Scott C. Howard

**Affiliations:** ^1^ Laboratory of Applied Statistics in the Health Sciences, School of Medicine University of Buenos Aires Buenos Aires Argentina; ^2^ Resonance Memphis Tennessee USA; ^3^ Division of Clinical Pharmacology, Toxicology & Therapeutic Innovation Children's Mercy Hospital Kansas City Missouri USA; ^4^ Department of Pediatrics University of Missouri – Kansas City School of Medicine Kansas City Missouri USA; ^5^ Division of Translational and Clinical Pharmacology Cincinnati Children's Hospital Medical Center Cincinnati Ohio USA; ^6^ Cancer and Blood Diseases Institute, Cincinnati Children's Hospital Medical Center Cincinnati Ohio USA; ^7^ Department of Pediatrics University of Cincinnati College of Medicine Cincinnati Ohio USA; ^8^ Pediatric Oncology and Hematology Service Vall d'Hebron University Hospital Barcelona Spain; ^9^ Pharmacy Service Reina Sofía University Hospital/Instituto Maimónides de Investigación Biomédica de Córdoba (IMIBIC)/University of Córdoba Córdoba Spain; ^10^ Department of Pediatrics, Pediatric Hematology Oncology Unit University of Milano‐Bicocca, IRCCS San Gerardo dei Tintori Monza Italy; ^11^ Sant Joan de Déu Hospital Barcelona Barcelona Spain; ^12^ Yeolyan National Hematology Center Yerevan Armenia

**Keywords:** acute kidney injury, biomarker, early methotrexate elimination half‐life, high‐dose methotrexate

## Abstract

**Introduction:**

High‐dose methotrexate (HDMTX) use can be limited by the development of acute kidney injury (AKI). Early AKI detection is paramount to prevent further renal injury and irreversible toxicities. This study sought to determine whether early elimination patterns of MTX would be useful as a biomarker of AKI in HDMTX treatment.

**Methods:**

This retrospective cohort study included two sites that collected ≥2 MTX levels within 16 h from completion of MTX infusion. Early levels were tagged and MTX elimination half‐life (t_½_) were calculated from combinations of two of three different early time periods. Receiver operating characteristic (ROC) curves were synthesized for each elimination t_½_ (biomarker) with respect to AKI and delayed methotrexate elimination (DME); the biomarker with the highest area under the ROC curve (AUC) was tested in a multiple variable logistic regression model.

**Results:**

Data from 169 patients who received a total of 556 courses of HDMTX were analyzed. ROC analysis revealed MTX elimination t_½_ calculated from the second and third time periods had the highest AUC for AKI at 0.62 (interquartile range [IQR] 0.56–0.69) and DME at 0.86 (IQR 0.73–1.00). After adjusting for age, sex, dose (mg/m^2^), infusion duration, HDMTX course, and baseline estimated glomerular filtration rate, it remained significant for AKI with an OR of 1.29 and 95% confidence interval of 1.03–1.65.

**Conclusion:**

Early MTX elimination t_½_ measured within 16 h of infusion completion was significantly associated with the development of AKI and serves as an early clearance biomarker that may identify patients who benefit from increased hydration, augmented leucovorin rescue, and glucarpidase administration.

## Graphical abstract

Methotrexate elimination half‐life calculated from two early measurements during high dose methotrexate treatment has the ability to discriminate between patients with and without acute kidney injury. This may serve as a valuable tool in identifying patients at risk of acute kidney and potentially facilitate timely interventions such as increased hydration, augmented leucovorin rescue, or glucarpidase administration.

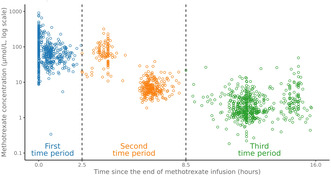

## 1 INTRODUCTION

High‐dose methotrexate (HDMTX) treatment has proven efficacy for acute lymphoblastic leukemia (ALL), lymphomas, and osteosarcoma. Its use may be limited, however, by the development of acute kidney injury (AKI) which can lead to delayed methotrexate elimination (DME) and subsequent toxicities related to prolonged MTX exposure.^1^ AKI is thought to be caused by precipitation of MTX and its metabolites in the renal tubules,^2^, ^3^ and occurs in 2%–33% of patients receiving HDMTX despite supportive care practices such as urine alkalinization to increase MTX solubility.^4^, ^5^, ^6^ AKI usually develops during the HDMTX infusion or shortly thereafter near the end of the steady‐state plasma concentration, with rising serum creatinine (S_cr_) indicating the deteriorating renal function.^7^ Because HDMTX‐induced AKI is often initially asymptomatic (non‐oliguric), early detection of AKI and subsequent DME is paramount to deliver appropriate intervention (e.g., augmented leucovorin rescue, glucarpidase) in order to prevent irreversible MTX‐related toxicities.^1^, ^7^, ^8^

An increase in S_cr_ of greater than 50% of baseline following the start of HDMTX infusion is the most widely used criterion for detecting AKI that warrants adjusting leucovorin dose or administration of glucarpidase. While serial measurement of S_cr_ is essential in monitoring renal function during and following HDMTX infusion, significant increases in S_cr_ may not be observed until 48–72 h after the initial renal insult resulting in delayed intervention.^9^ Further, nonrenal factors such as muscle mass and age, and renal factors such as renal reserve and tubular secretion of creatinine render the utility of S_cr_ for early detection of AKI less than optimal. To address these limitations, several biomarkers for early detection of AKI have been investigated, with cystatin C and neutrophil gelatinase‐associated lipocalin (NGAL) showing promise in patients treated with HDMTX.^3^, ^10^, ^11^, ^12^ Use of new biomarkers to monitor renal function, however, would require not only additional laboratory testing that may be cost prohibitive but also more validation through clinical trials in patients of varying ages, diagnoses, and HDMTX infusion durations.

In addition to S_cr_, serial plasma MTX concentrations (MTXc) are routinely assessed following HDMTX infusion to monitor renal elimination of MTX. Current guidelines for treating severe AKI and DME recommend intravenous glucarpidase administration between 48 and 60 h following the start of a 24‐h HDMTX infusion based on MTXc at 36‐, 42‐, or 48‐h depending on the MTX dose.^13^ However, these time points may be too late to prevent permanent toxicities in cases where significant AKI develops earlier during infusion. The National Comprehensive Cancer Network Guidelines in Oncology® (NCCN Guidelines®) recommend glucarpidase administration when MTXc is two standard deviations above the mean expected MTXc as determined by MTXPK.org.^14^ MTXPK.org is a web‐based clinical decision support tool that has been validated to assess HDMTX pharmacokinetics; it utilizes the patient's demographic characteristics, S_cr_, and real‐time MTXc to predict MTX elimination.^15^ Plasma MTXc is usually measured around the time of infusion completion and at 18–24‐h intervals thereafter up to 72 h after infusion completion to monitor MTX clearance.^13^, ^16^, ^17^ Earlier MTX measurements are not routinely obtained in clinical practice; however, recent evidence from pig models have shown that MTX levels collected within 4 h of completing a HDMTX infusion were able to predict the development of AKI when a slower exponential decline in MTXc was detected.^18^ The pig model of early MTX elimination may translate to similar findings in humans using MTX measurements collected within 12 h of HDMTX infusion completion and would allow for earlier detection and intervention for AKI and DME.

This study was an ad hoc investigation that commenced following a quality review of the HDMTX European Registry (clinicaltrials.gov NCT05899751), henceforth referred to as “Registry”. Review of the Registry database revealed a significant number of HDMTX courses in which MTX level measurements were collected much earlier than usually performed in clinical practice and was determined that it provided sufficient data to investigate the potential usefulness of early MTX levels. This study was conducted to determine whether early elimination of MTX soon after infusion completion is useful as an early clearance biomarker of AKI in HDMTX treatment.

## 2 METHODS

### 2.1 Study population

The study population was drawn from the ongoing Registry which consists of patients who had received HDMTX treatment across 10 distinct European clinical sites. The inclusion criteria for the Registry encompass patients of any age, any type of cancer diagnosed from January 1, 2001 to June 30, 2021, receipt of HDMTX chemotherapy (defined as a dose ≥500 mg/m^2^ of body surface area infused over 1–36 h), and availability of medical records for review. The Registry employed convenience sampling with priority given to the most recently diagnosed patients from each clinical site.

For this retrospective cohort study, an ad hoc subset of patients originating from two separate sites in Spain which had completed data entry and site closeout were selected as they had collected at least two MTXc measurements within 16 h of HDMTX infusion completion in accordance with their institutional protocols and practices (Appendix S1). Both sites employed immunoassay to measure MTXc. Notably, no additional inclusion or exclusion criteria were imposed for the patients selected from these two sites.

### 2.2 Outcome variables

This study defined two outcome variables to assess HDMTX treatment effects: AKI and DME. The *Acute Kidney Injury Network* (*AKIN*) criteria was employed to determine the incidence of AKI. The AKIN criteria categorize AKI into three grades: Grade 1, an increase in S_cr_ by at least 0.3 mg/dL within 48 h *OR* an increase of 1.5–1.9 times baseline within 7 days; Grade 2, S_cr_ increase of 2.0–2.9 times baseline; and Grade 3, S_cr_ increase ≥ three times baseline *OR* S_cr_ ≥4.0 mg/dL *OR* the initiation of renal replacement therapy.^19^ For this study, severe AKI was defined as meeting the AKIN criteria for Grade 2 or 3 at each course of HDMTX treatment. For DME, two distinct criteria were employed. The *micromolar* criterion determined the presence of DME if MTXc >1 μM at any time point after 42 h from the start of HDMTX infusion, regardless of the MTX infusion duration.^20^ The *standard deviation* (*SD*) criterion determined DME to be present if MTXc >2 SD from the population mean as determined by MTXPK.org at 42‐ or 48‐h from the start of infusion.^14^ Briefly, MTXPK.org is a validated pharmacokinetic modeling tool that simulates MTXc based on available data points that did not meet the criteria for early levels, and allows for a standardized and clinical relevant metric for assessing DME.^15^ All MTXc measurements taken after 16 h of infusion completion were input to the model to predict MTXc at 42‐ or 48‐h from the start of infusion, which were subsequently applied to the aforementioned DME criteria.

### 2.3 Explanatory variables

A set of potential explanatory variables were calculated to capture the dynamics of MTX levels early during the HDMTX treatment. Firstly, the temporal progression of MTX levels was categorized into three distinct time periods based on visual exploration and prior clinical expertise. The time periods were designated based on when the MTX levels were measured following the completion of infusion: the “first” time period, at less than 2.5 h; “second” time period, between 2.5 and 8.5 h; and “third” time period, between 8.5 and 16 h. If two or more measurements occurred in the same period, the closest to the end of MTX infusion was used. In cases of 24‐h infusions, if a level was drawn within 5 h before the end of infusion it was imputed as hour 0. Secondly, all the combinations of MTX levels collected from two of the three time periods (i.e., “first” and “second”, “first” and “third”, and “second” and “third”) were used to calculate two distinct early MTX clearance biomarkers: elimination half‐life and slope (rate of MTXc decline). An exponential decay equation was used to calculate the elimination half‐life of MTX for each combination of time periods and the slope of a line connecting the two selected time periods was calculated for each combination to determine the rate of decline of MTXc (Appendix S2).

### 2.4 Covariables

Demographic characteristics of patients and relevant clinical information including cancer type, HDMTX dose and infusion duration, number of courses, estimated glomerular filtration rate (eGFR), and S_cr_ and MTX levels were collected. The Chronic Kidney Disease Epidemiology Collaboration (CKD‐EPI) equation was used to determine the eGFR for patients older than 16 years, and the revised Schwartz formula for those younger.^21^, ^22^

### 2.5 Statistical analysis

Descriptive statistics were used to summarize the demographic characteristics of the patients and relevant clinical information. Continuous variables were presented as median and interquartile range (IQR). Categorical variables were summarized using absolute frequencies and percentages.

Each early MTX clearance biomarker (i.e., elimination half‐life and slope) calculated from the combinations of MTX levels at different time periods underwent receiver operating characteristic (ROC) analysis. The ROC curve is a graphical representation of the trade‐off between sensitivity and specificity for a given test or biomarker. The area under the ROC curve (AUC) and its corresponding confidence interval for AKI and DME were calculated for each biomarker to provide a measure of its discriminatory power.^23^, ^24^ A statistically significant AUC is one where the confidence interval excludes the null hypothesis value of 0.5, which represents a biomarker with no discriminatory power (i.e., equivalent to random chance). The biomarker with the highest AUC in relation to AKI was selected for further analysis.

The selected biomarker was subsequently tested in a multiple variable model, accounting for a priori selected potential confounding factors under a causal inferential framework: age (years), sex, dose (mg/m^2^), infusion duration (short [4 h] or long [24 h] infusions), HDMTX course (numeric discrete), and baseline eGFR (mL/min/1.73m^2^) (Appendix S3).^25^, ^26^, ^27^ This adjustment was carried out using a logistic regression model with a significance level (alpha) set at 0.05. Using a likelihood‐ratio test, a comparison was made between two nested logistic regression models: one incorporating the predictor (biomarker) and another without it to assess whether the inclusion of the biomarker significantly improves the model fit and contributes information beyond the other variables included in the model.

Timing of AKI occurrence was analyzed, and a sensitivity analysis was conducted by excluding any events occurring before or simultaneously with the calculation of the early clearance biomarker. This analysis focused on the best‐performing biomarker pair to ensure accurate assessment of its performance.

### 2.6 Ethical considerations

Approval from the Comité de Ética de la Investigación Provincial de Córdoba, Hospital Universitario Reina Sofía, Universidad de Córdoba, Spain and the Comité de Ética de la Investigación, Hospital Universitari Vall d'Hebron, Universitat Autònoma de Barcelona, Spain were granted prior to initiation of data collection. Waivers of consent were provided by both IRBs.

## 3 RESULTS

### 3.1 Study population, methotrexate measurements, and outcomes

**FIGURE 1:**
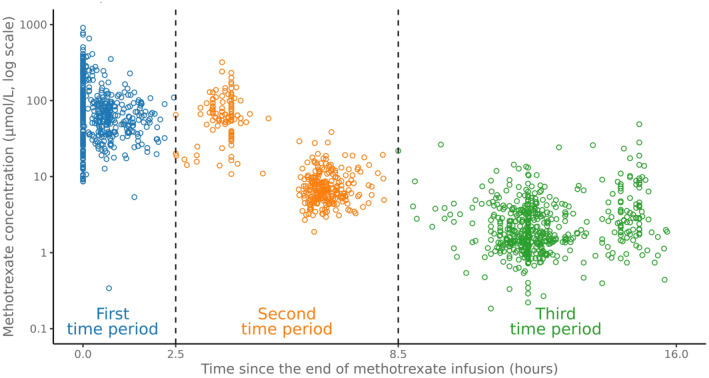
Time‐period classification for methotrexate measurements following completion of infusion.

The study population consisted of 169 patients who had received a total of 556 courses of HDMTX. The median age of the patients was 10.3 years (IQR 4.2–37.3) and 114 (67.5%) were under the age of 18 at diagnosis. Two‐thirds (66.9%) were diagnosed with B‐lineage ALL (Table 1). Of the 556 available courses, 329 (59.2%) had MTX measurements obtained at all three designated time periods, and 83% of the courses had received long infusions (24 h). Courses with MTX levels collected at only the first and third time periods were those of older patients, who also received a lower dose of MTX (Table 2). Median collection time for the first, second, and third early MTX measurements were at 0.17 (IQR 0.00–0.68), 6.28 (IQR 4.00–6.62), and 12.00 (IQR 11.80–12.80) hours after infusion completion, respectively (Figure 1). Median MTXc were 59.6 μM (IQR 34.1–84.6), 6.8 μM (IQR 5.2–10.0), and 1.7 μM (IQR 1.2–3.2), respectively, for the three time periods of the 24‐h infusions and 247.2 μM (IQR 196.2–313.3), 66.5 μM (IQR 54.3–100.0), and 3.1 μM (IQR 2.0–7.2), respectively, for the three time periods of the 4‐h infusions. Among the total 556 HDMTX courses, AKI developed in 168 (30.2%) courses, DME by micromolar criterion in 215 (38.7%), and DME by SD criterion in 38 (6.8%) courses (Table 2).

**TABLE 1:** Patient characteristics (*n* = 169).

Age at diagnosis, median years (IQR)		10.3 (4.2–37.3)
Age under 18 years, *n* (%)		114 (67.5%)
Sex, *n* (%)	Female	67 (39.6)
	Male	102 (60.4)
Diagnosis, *n* (%)	ALL, not specified	3 (1.8)
	ALL, B‐cell lineage	113 (66.9)
	ALL, T‐cell lineage	14 (8.3)
	NHL	39 (23.1)
Site, *n* (%)	A	89 (52.7)
	B	80 (47.3)

Abbreviations: ALL, acute lymphoblastic leukemia; IQR, interquartile range; NHL, non‐Hodgkin lymphoma (includes primary central nervous system lymphoma).

**TABLE 2:** Clinical characteristics of HDMTX courses by methotrexate level measurement time periods.

		Total courses (*n* = 556)	First and second time periods (*n* = 340)	First and third time periods (*n* = 533)	Second and third time periods (*n* = 341)
Age at HDMTX infusion, median years (IQR)		9.6 (4.4–37.7)	6.6 (3.9–15.5)	9.7 (4.4–37.7)	6.6 (4.0–15.5)
Sex, *n* (%)	Female	240 (43.2)	139 (40.9)	228 (42.86)	139 (40.8)
	Male	316 (56.8)	201 (59.1)	305 (57.2)	202 (59.2)
MTX dose, median mg/m^2^ (IQR)		4834 (2849–4984)	4921 (3008–4993)	4833 (2845–4982)	4921 (3008–4991)
Baseline creatinine, median mg/dL (IQR)		0.4 (0.3–0.6)	0.4 (0.3–0.5)	0.4 (0.3–0.6)	0.3 (0.3–0.5)
Baseline eGFR, mL/min/1.73m^2^ (IQR)		132.7 (107.4–159.2)	140.2 (110.1–165.6)	132.6 (107.2–159.4)	140.5 (111.9–165.2)
Infusion length, *n* (%)	Long (24 h)	461 (82.9)	249 (73.2)	442 (82.9)	252 (73.9)
	Short (4 h)	95 (17.1)	91 (26.8)	91 (17.1)	89 (26.1)
AKI, *n* (%)	No AKI	388 (69.8%)	235 (69.1%)	372 (69.8%)	235 (68.9%)
	Grade 1	127 (22.8%)	79 (23.2%)	124 (23.3%)	78 (22.9%)
	Grade 2	36 (6.5%)	24 (7.1%)	32 (6.0%)	26 (7.6%)
	Grade 3	5 (0.9%)	2 (0.6%)	5 (0.9%)	2 (0.6%)
DME *n* (%)	SD	38 (6.8%)	15 (4.4%)	35 (6.6%)	16 (4.7%)
	μM	215 (38.7%)	130 (38.2%)	204 (38.3%)	133 (39.0%)
Measurement time periods, *n* (%)	1st and 2nd	11 (2.0)	11 (3.2)	0	0
	1st and 3rd	204 (36.7)	0	204 (38.3)	0
	2nd and 3rd	12 (2.2)	0	0	12 (3.5)
	1st, 2nd, and 3rd	329 (59.2)	329 (96.8)	329 (61.7)	329 (96.5)
Site, *n* (%)	A	285 (51.3)	92 (27.1)	278 (52.2)	93 (27.3)
	B	271 (48.7)	248 (72.9)	255 (47.8)	248 (72.7)

Abbreviations: AKI, acute kidney injury; DME, delayed MTX elimination; eGFR, estimated glomerular filtration rate; IQR, interquartile range; MTX, methotrexate; SD, standard deviation criterion; μM, micromolar criterion.

### 3.2 ROC analysis of the early clearance biomarkers

**FIGURE 2:**
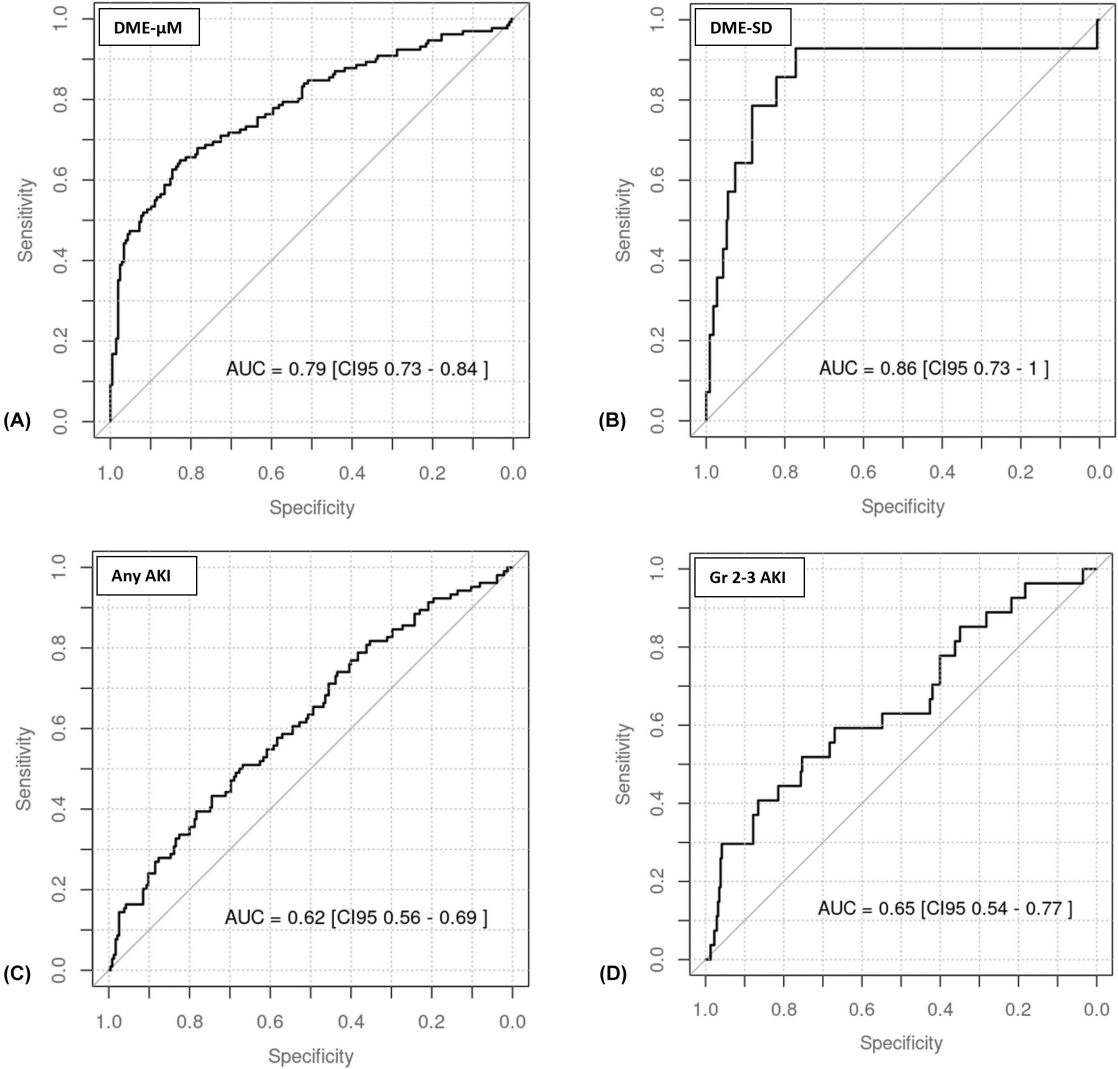
Receiver operating characteristic (ROC) curves for methotrexate elimination half‐life calculated from the second and third time‐period measurements. (A) DME by micromolar criterion; (B) DME by standard deviation criterion; (C) AKI of any grade; (D) AKI grades 2–3.

ROC analysis revealed the MTX elimination half‐life obtained between the second and third time periods to have the highest AUC for AKI compared to the other biomarkers using different time‐period combinations. It was the best performing early clearance biomarker for any grade AKI with an AUC of 0.62 (IQR 0.56–0.69) and 0.65 (IQR 0.54–0.77) for severe AKI; it also discriminated DME with an AUC of 0.79 (IQR 0.73–0.84) for the micromolar criterion, and 0.86 (IQR 0.73–1.00) for the SD criterion (Table 3, Figure 2). The median elimination half‐life obtained between the second and third time periods were 2.95 h (IQR 2.51–3.47) and 2.51 h (IQR 2.34–2.80) for the 24‐ and 4‐h infusions, respectively.

**TABLE 3:** Area under the curve (AUC) of early clearance biomarkers (slope and elimination half‐life).

Biomarker (*n*)	T1	T2	DME–μM AUC [IQR]	DME–SD AUC [IQR]	AKI any grade AUC [IQR]	AKI grade 2–3 AUC [IQR]
Slope 1–2 (340)	First	Second	0.57 [0.51–0.63]	0.55 [0.42–0.69]	0.52 [0.45–0.58]	0.59 [0.50–0.68]
Slope 1–3 (533)	First	Third	0.56 [0.51–0.61]	0.57 [0.46–0.68]	0.54 [0.49–0.59]	0.60 [0.52–0.69]
Slope 2–3 (341)	Second	Third	0.49 [0.43–0.55]	0.59 [0.43–0.75]	0.51 [0.45–0.58]	0.57 [0.46–0.67]
Half‐life 1–2 (340)	First	Second	0.57 [0.51–0.64]	0.83 [0.72–0.95]	0.50 [0.43–0.57]	0.58 [0.45–0.72]
Half‐life 1–3 (533)	First	Third	0.66 [0.62–0.71]	0.93 [0.90–0.96]	0.57 [0.51–0.63]	0.64 [0.52–0.75]
Half‐life 2–3 (341)	Second	Third	0.79 [0.73–0.84]	0.86 [0.73–1.00]	0.62 [0.56–0.69]	0.65 [0.54–0.77]

Abbreviations: AKI, acute kidney injury; DME–μM, delayed methotrexate elimination by micromolar criterion; DME–SD, DME by standard deviation criterion; T1, time‐period 1 of two time‐point combination; T2, time‐period 2 of two time point of combination.

### 3.3 Multiple variable model

After adjusting for age, sex, dose, infusion duration, HDMTX course, and baseline eGFR, the elimination half‐life between the second and third time periods remained statistically significant for AKI with an OR of 1.29 and 95% CI [1.03, 1.65] and a *p*‐value of 0.031. The comparison of the model with and without this early clearance biomarker using a likelihood ratio‐test showed that the biomarker improved the overall performance with a *p*‐value of 0.006 (Appendix S4).

### 3.4 Sensitivity analysis

Since creatinine measurements were conducted according to institutional protocols, AKI could have theoretically occurred before the early clearance biomarker was calculated. Ultimately, five cases of AKI (4.7%) were identified 12 h after the end of infusion, while 101 cases (95.2%) were observed after 20 h following infusion completion. An ad hoc sensitivity analysis excluding the five cases of earlier AKI occurrence revealed that the early clearance biomarker's performance remained consistent using the half‐life obtained between the second and third time periods. For any grade of AKI, the biomarker yielded an AUC of 0.61 (IQR 0.55–0.68) and 0.64 (IQR 0.52–0.76) for severe AKI. It also discriminated DME with an AUC of 0.78 (IQR 0.72–0.83) for the micromolar criterion and 0.84 (IQR 0.67–1.00) for the SD criterion. In the multivariable logistic regression model, the biomarker remained statistically significant for AKI, with an odds ratio (OR) of 1.32 (95% CI [1.04, 1.72]) and a *p*‐value of 0.027.

## 4 DISCUSSION

This study aimed to determine whether early elimination of MTX soon after infusion completion is useful as an early clearance biomarker of AKI in HDMTX treatment. Early MTX elimination half‐life calculated using the second (4–6 h) and third (12 h) time periods was associated with the development of AKI. This finding held after adjusting for potential confounding factors. As this biomarker is based on the rate of elimination between two early time points, it may be useful in detecting AKI much earlier than can be detected by increases in S_cr_.^18^ Early detection of patients at risk of developing AKI may allow for prompt intervention to prevent further renal damage. Clinically, this finding could inform decisions regarding supportive measures such as adjusting fluids, optimizing diuresis, and administering leucovorin or glucarpidase. Consensus guidelines suggest that the optimal timing for glucarpidase delivery is between 48 and 60 h from the start of HDMTX infusion, as beyond this point, life‐threatening toxicities may not be preventable.^13^ By identifying patients at risk sooner, healthcare providers can implement protective strategies like increased hydration, augmented leucovorin rescue, and glucarpidase in the early hours, when they have the potential to make the most difference. This proactive approach could mitigate the progression of renal injury, reduce the duration of other HDMTX‐related toxicities, and improve patient outcomes by preventing the development of severe AKI.

Current practice varies, but most clinical trials or hospital protocols do not require MTXc testing early after infusion completion. Past studies on HDMTX suggest that the high MTXc during the first 24 h prior to leucovorin rescue produces minimal toxicity.^28^ Further, MTX levels obtained during the first 24 h following MTX delivery is considered not to be predictive of subsequent high MTXc and thus should not be used as the basis for glucarpidase administration.^29^ The findings of this study challenge the prevailing notion that AKI can only be effectively detected after significant renal damage has occurred, and highlight that the early clearance biomarker for methotrexate allows for early detection of impending AKI and prompt intervention. Furthermore, MTX elimination varies widely among individuals which leads to the wide range of concentrations (1000‐fold among patients receiving the same dose).^30^ Therefore, identifying early elimination rates specific to the individual patient may be instrumental for detecting potential AKI in those at risk. Under this concept, the two institutions from which this data originated have been collecting these early MTX levels to proactively provide increased hydration and/or leucovorin rescue based on the patients' individual pharmacokinetics, that is, rate of MTX elimination (Appendix S1).

A key strength of this study is the comprehensive approach employed to select and evaluate the MTX early clearance biomarker. The ROC analysis was initially employed to evaluate and select the most promising biomarker among different candidates. After the selection, it was used to establish the discriminative power of the MTX elimination half‐life as an early clearance biomarker, providing insight into the trade‐off between sensitivity and specificity. Given that this type of analysis takes into account only one variable, the analysis was augmented with a multiple variable logistic regression model to strengthen the claim after taking into account different confounding variables. Additionally, this study's observational nature based on real‐world clinical data and supported by a careful and systematic method of data collection, enhanced the relevance and applicability of the results.

There are several limitations to this study. The number of time periods for collection of MTX levels were variable among the patients and the actual timing of the collections were nonuniform. This is inherent to the retrospective nature of the study as well as the data originating from two distinct sites. It is notable, however, that the first time period (less than 2.5 h from completion) mirrors the MTX measurement that is usually performed after completion of infusion, and the third time period (between 8.5 and 16 h following completion) is similar to that taken at usually 36–42 h from start of a 24‐h infusion. The second time period of collection (between 2.5 and 8.5 h following end of infusion), however, is relatively unique to this study population with the collection times clustered at 4 and 6 h (Figure 1). As the elimination half‐life estimated from the MTX levels between the second and third time periods were found to be associated with the development of AKI and DME, this second time‐period level may serve as an important measure to collect in clinical practice. The precise timing for when this measurement should take place remains to be determined. Secondly, as the patients from the two institutions underwent early MTX level monitoring to modify their hydration, those with higher MTXc likely received increased hydration to prevent AKI which could have reduced the discriminatory effect of the early clearance biomarker. Despite this potential limitation, the results of the multiple variable analyses suggest that the discriminatory power of the biomarker still holds. The under‐representation of older adults is another significant limitation. The study population consisted predominantly of children and young adults who likely have better renal function and lower incidence of AKI than older adults that may present with different MTX pharmacokinetics during the same period following infusion completion.^31^ Nevertheless, young patients receiving HDMTX treatment generally have fewer comorbidities, which allows for a more controlled examination of MTX elimination and AKI risk without the confounding effects of multiple chronic conditions. The lack of patients diagnosed with osteosarcoma and relatively limited number of patients with primary central nervous system lymphoma (PCNSL) also limits the study results from being generalized to individuals receiving higher doses of MTX, shorter infusion duration, and concomitant use of other nephrotoxic drugs routinely used for treatment in these conditions.^32^, ^33^

## 5 CONCLUSIONS

An early clearance biomarker based on the elimination half‐life of MTX estimated between 2.5 and 16 h after the end of HDMTX infusion was associated with increased odds of AKI and identified patients who developed AKI and DME. Using individualized pharmacokinetics of early MTX elimination soon after infusion completion as an early clearance biomarker to predict the potential development of AKI may help prevent nephrotoxicity and improve the safety and efficacy of HDMTX. Clinical trials incorporating measurement of early MTX levels in older adults and other cancers (e.g., osteosarcoma) are needed to validate the early clearance biomarker in other populations receiving HDMTX treatment. Additionally, further investigations are needed to determine a specific threshold of the early clearance biomarker to incorporate it into clinical practice.

## AUTHOR CONTRIBUTIONS

**Nicolás Tentoni:** Conceptualization (equal); data curation (equal); formal analysis (lead); methodology (lead); project administration (lead); visualization (lead); writing – original draft (equal). **Miriam Hwang:** Project administration (supporting); writing – original draft (equal). **Gabriela Villanueva:** Conceptualization (supporting); supervision (supporting). **Ryan Combs:** Data curation (equal); formal analysis (supporting); funding acquisition (equal); project administration (supporting); supervision (equal); validation (equal). **Jennifer Lowe:** Data curation (equal); funding acquisition (equal); project administration (supporting). **Laura B. Ramsey:** Conceptualization (supporting); methodology (supporting). **Zachary L. Taylor:** Conceptualization (supporting); methodology (supporting). **Thais Murciano Carrillo:** Conceptualization (supporting); investigation (equal); methodology (supporting). **María Dolores Aumente:** Conceptualization (supporting); investigation (equal); methodology (supporting). **Teresa López‐Viñau:** Conceptualization (supporting); investigation (equal); methodology (supporting). **Carmelo Rizzari:** Conceptualization (equal); supervision (supporting). **Scott C. Howard:** Conceptualization (equal); formal analysis (supporting); funding acquisition (equal); methodology (equal); supervision (lead); validation (equal); visualization (supporting).

## CONFLICT OF INTEREST STATEMENT

L. Ramsey, S. Howard: Consultant for BTG Pharmaceuticals. N Tentoni, M Hwang, G. Villanueva, R Combs, J Lowe, S Howard: Resonance received grant funding from BTG Pharmaceuticals. L. Ramsey: Research funded by BTG Pharmaceuticals. C. Rizzari: Sponsored speaker and member of advisory boards of Clinigen, SERB, Servier, Jazz Pharmaceuticals and Amgen. All remaining authors have no conflict of interests.

## Supporting information


Appendices S1–S4.

## Data Availability

The data that support the findings of this study are available from the corresponding author upon reasonable request.
