# Supplementary material for: Early therapeutic drug monitoring of methotrexate and its association with acute kidney injury: A retrospective cohort study
Source: Cancer Med. 2024 Sep 10;13(17):e70176. doi: 10.1002/cam4.70176 (PMC11386298; doi:10.1002/cam4.70176)
Supplement: Supplementary file 1 — Appendices S1–S4. [file CAM4-13-e70176-s001.zip › Appendices_mtx_drug_monitor_aki_clean.docx]

**Appendix 1 – Institutional protocols for Sites A and B for HDMTX infusion**

- Site A
- 24-hour infusion

If MTXc > 100 µM (by immunoassay) at 12 hours after the start of infusion, hydration is increased. Leucovorin dose adjustment was determined by the estimated MTXc at 42 hours using Bayesian estimation based on the MTXc at 2, 12, 23, and 36 hours from the start of infusion.

- 4-hour infusion

Leucovorin dose adjustment is determined by the estimated MTXc at 24 hours using Bayesian estimation based on the MTXc at 4 (end of infusion), 8, and 19 hours from the start of infusion.

- Site B
- Before 2020

MTXc measured (by immunoassay) at 24, 30 and 36 hours after the start of infusion with leucovorin rescue beginning at 36 hours. Leucovorin dose adjustment according to the Bleyer nomogram and increased hydration at 24 hours if MTXc > 150 µM.

- After 2020

MTXc measured (by immunoassay) at 24 hours after the start of infusion.

If MTXc > 120 µM and/or serum creatinine (S_cr_) increases 1.5 times baseline, hydration is increased and repeat MTXc measurement taken at 36 hours and leucovorin rescue initiated.

If the MTXc and S_cr_ at 24 hours are normal, MTXc measurement taken and leucovorin rescue at 42 hours.

**Appendix 2**

- Exponential decay equation


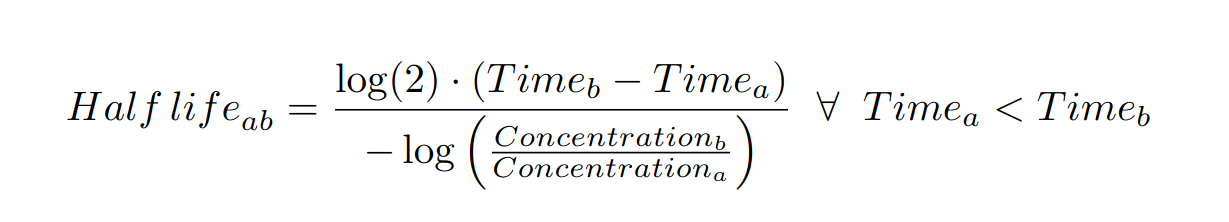


- Slope equations


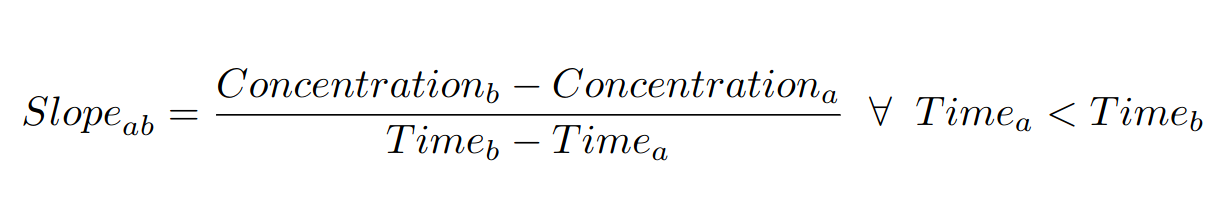


**Appendix 3 – Variable selection for the multiple variable model**

The variables were selected because they establish a backdoor pathway between the causal relationship of early methotrexate elimination and AKI. As the volume of distribution is a potential confounder, body surface area and methotrexate dose were combined into a singer variable (dose/m^2^).

Figure S1. Causal directed acyclic graph


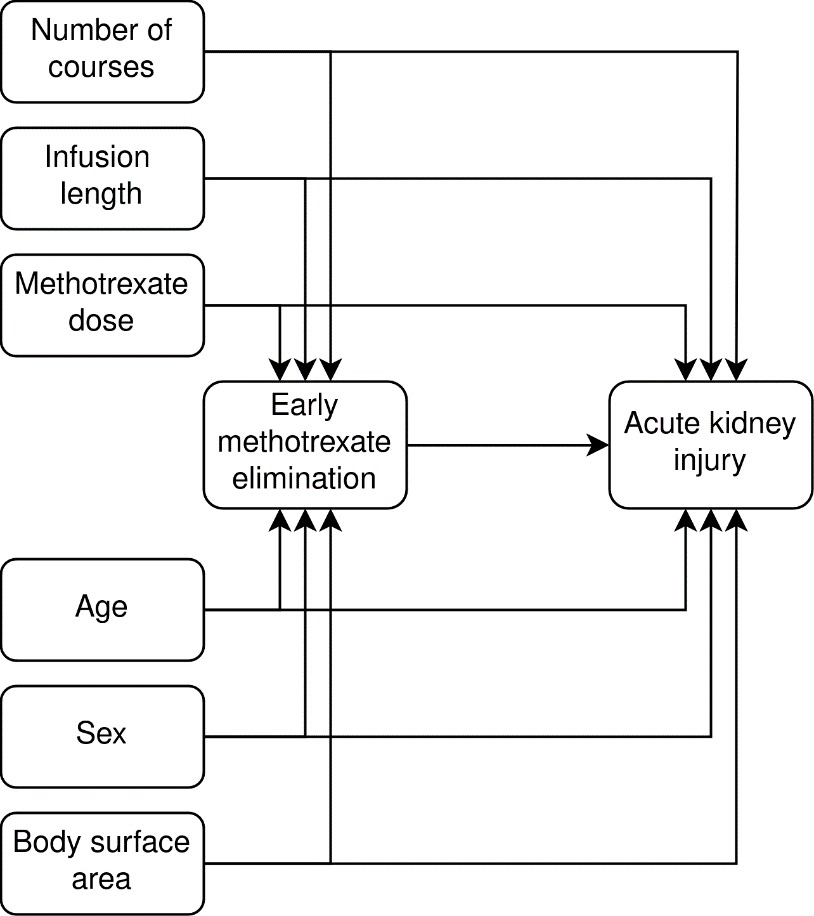


**Appendix 4 – Logistic regression results and diagnostics**

| **Variable** | **OR [95% CI]** | **Beta** | **Beta SE** | **p-value** |
| --- | --- | --- | --- | --- |
| Early MTX elimination half-life | 1.29 [1.03, 1.65] | 2.16 | 0.12 | 0.031 |
| eGFR (ml/min/1.73m^2^) | 1.01 [1.00, 1.01] | 1.72 | 0.00 | 0.086 |
| Age (years) | 1.01 [0.99, 1.04] | 1.19 | 0.01 | 0.233 |
| MTX dose (mg/m^2^) | 1.00 [1.00, 1.00] | 1.76 | 0.00 | 0.078 |
| Short infusion duration (4-hour) | 0.94 [0.30, 2.77] | -0.12 | 0.56 | 0.904 |
| Male sex | 0.89 [0.54, 1.47] | -0.46 | 0.26 | 0.644 |
| Course number | 0.73 [0.59, 0.90] | -2.99 | 0.10 | 0.003 |
| (Intercept) | 0.04 [0.00, 0.45] | -2.52 | 1.23 | 0.012 |

Hosmer-Lemeshow goodness-of-fit test

χ^2^: 4.4

Degrees of freedom: 8

p-value: 0.8
