# Supplementary figures and images for: Early therapeutic drug monitoring of methotrexate and its association with acute kidney injury: A retrospective cohort study
Source: Cancer Med. 2024 Sep 10;13(17):e70176. doi: 10.1002/cam4.70176 (PMC11386298; doi:10.1002/cam4.70176)

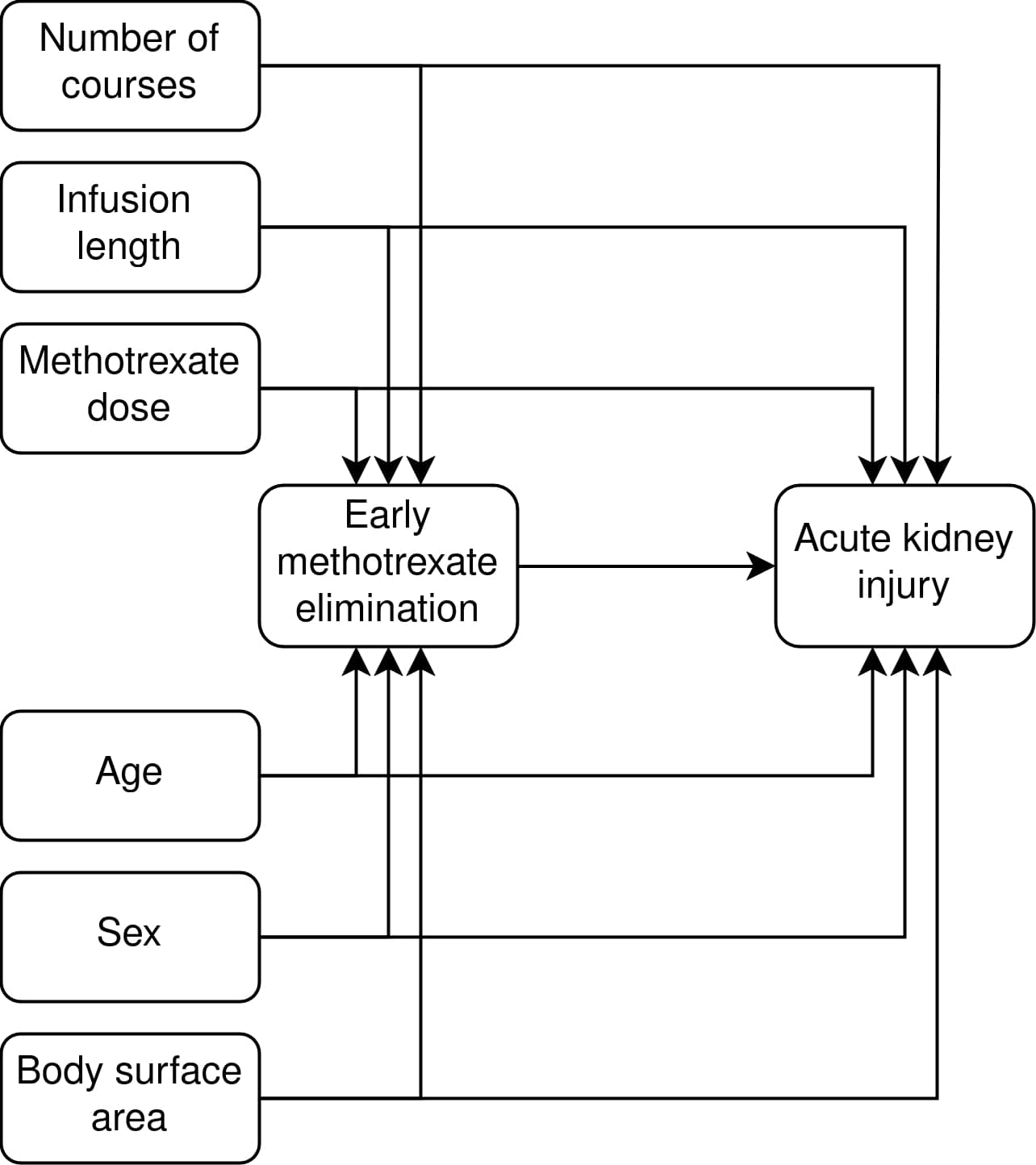

Supplement: Supplementary file 1 — Appendices S1–S4. [file CAM4-13-e70176-s001.zip › Appendix_3_figure.jpg]
